# The influence of intercropping *Paris polyphylla* with *Polygonatum cyrtonema* or *Ganoderma lucidum* on rhizosphere soil microbial community structure and quality of *Paris polyphylla*

**DOI:** 10.3389/fmicb.2025.1697736

**Published:** 2025-11-06

**Authors:** Hailan Su, Penghui Liao, Hong Chen, Fengfang Lin, Meixia Zheng, Yuqing Niu, Wei Ye, Fanghua Mao, Yujing Zhu, Yanghui Fang, Yanming Zhu

**Affiliations:** 1 Institute of Crop Sciences, Fujian Academy of Agricultural Sciences (Fujian Germplasm Resources Center), Fuzhou, China; 2 Fujian Forestry Science and Technology Extension General Station, Fuzhou, China; 3 Sanming Academy of Agricultural Sciences, Sanming, China

**Keywords:** *Paris polyphylla*, intercropping, rhizospheric microorganism, soil physicochemical properties, polyphyllin

## Abstract

**Introduction:**

Prolonged monoculture cultivation of *Paris polyphylla* has been linked to challenges such as disruption of rhizosphere soil microbial balance and deterioration in crop quality. Intercropping has emerged as a viable strategy to address these issues.

**Methods:**

In the present study, monoculture of *P. polyphylla* (PP) served as the control, while two intercropping systems were implemented: *P. polyphylla* with *Polygonatum cyrtonema* (PPPC) and *P. polyphylla* with *Ganoderma lucidum* (PPG). The objective was to evaluate the *P. polyphylla* quality, soil physicochemical properties, and the structure of the microbial community.

**Results:**

Findings revealed that the PPG intercropping system significantly increased available potassium levels by 50.28% and enhanced the abundance of *Trichoderma* by 3,022% via the *G. lucidum* network. These alterations were associated with improvements in *P. polyphylla* yield (51.30%), polyphyllin VII content (34.16%), and total polyphyllin content (30.59%). Conversely, the PPPC system promoted the enrichment of *Cupriavidus* and nitrogen-fixing bacteria such as *Hyphomicrobiales*, leading to a 26.78% rise in available phosphorus and a 20.00% increase in polyphyllin II content in *P. polyphylla*. Both intercropping approaches markedly elevated the abundance of Basidiomycota, with the PPPC system further enriching functional microbial taxa including Glomeromycota and *Saitozyma*.

**Discussion:**

Collectively, these results demonstrate that intercropping *P. polyphylla* with either *P. cyrtonema* (PPPC) or *G. lucidum* (PPG) enhances soil physicochemical attributes, optimizes the composition of rhizosphere microbial communities, and positively influences the accumulation and yield of bioactive compounds.

## 1 Introduction

*Paris polyphylla* is recognized as a nationally designated second-class rare medicinal resource. Its rhizome contains polyphyllins I, II, and VII, which constitute the principal bioactive compounds in traditional Chinese patent medicines renowned for their heat-clearing and detoxifying effects (Wu et al., 2017; Liu L. L. et al., 2025). In recent years, the scale of artificial cultivation of *P. polyphylla* has expanded steadily. Nevertheless, prolonged monoculture or continuous cropping of traditional Chinese medicinal plants frequently results in a triad of challenges, including soil microbial imbalance, nutrient depletion, and a consequent decline in crop quality (Li M. L. et al., 2025). Recent studies conducted in extensive cultivation areas have demonstrated that the species' 5–7 years growth cycle, combined with low root exudation, leads to a marked decrease in available potassium within the rhizosphere, which declines to 93 mg·kg^−1^ after 4 to 5 years of continuous planting—approximately 55% of the levels observed in newly cultivated soils (Xian et al., 2022; Su et al., 2024). Concurrently, the relative abundance of Basidiomycota fungi diminished significantly from 18% to 6%, while potentially pathogenic taxa such as *Fusarium* proliferated. These alterations were correlated with a reduction exceeding 15% in polyphyllin VII content (Xian et al., 2022; Su et al., 2024).

For crops with a long growth period, intercropping has emerged as an effective strategy to alleviate these issues. This approach has been systematically investigated in model plant and food crop systems. For example, the pineapple-banana rotation promotes the proliferation of *Pseudomonas* via root-exuded carboxylic acids, resulting in a increase in soil available phosphorus and a significant suppression of Fusarium wilt disease (Yang et al., 2023). Similarly, wheat-fava bean intercropping leverages the nitrogen-fixing capacity and flavonoid secretion of fava bean to restructure bacterial-fungal interaction networks, thereby improving both yield and soil health (Li et al., 2016). Within the context of medicinal plants, continuous cultivation of ginseng over 2 years has been linked to reductions in soil available phosphorus as well as declines in quality and yield (Rao et al., 2025). Intercropping with *Asari Radix* et *Rhizoma*, which emits volatile oils capable of inhibiting ginseng pathogens and promoting phosphate-solubilizing bacteria, has been demonstrated to increase saponin content in ginseng (Feng et al., 2008; Wei et al., 2024). Collectively, these examples highlight the effectiveness of disrupting the deleterious microecological cycles induced by monoculture through integrated systems involving companion plants, functional microorganisms, and soil interactions.

Two medicinal plants, *Polygonatum cyrtonema* and *Ganoderma lucidum*, share similar ecological characteristics with *P. polyphylla*. All three species are shade- tolerant plants and exhibit complementary ecological traits. Notably, *P. cyrtonema* enhances soil phosphorus and potassium availability via root-secreted organic acids (Lu et al., 2025; Chen et al., 2002), whereas *G. lucidum*, a white rot fungus, decomposes lignocellulose through its mycelial network, thereby releasing bioavailable potassium and secreting polysaccharides that induce plant resistance (Fang et al., 2024). However, systematic investigations remain limited regarding whether the tripartite “plant-fungus-plant” interactions among these species can selectively reconstruct the rhizosphere microecology of *P. polyphylla* and improve the quality of its medicinal components. This study focuses on *P. polyphylla*, which is notably affected by continuous cropping obstacles. Three cultivation regimes were established: monoculture of *P. polyphylla* (PP), intercropping with *P. cyrtonema* (PPPC), and intercropping with *G. lucidum* (PPG). The present research examines changes in agronomic traits, active ingredient concentrations, rhizosphere soil physicochemical properties, and microbial community composition of *P. polyphylla*. Furthermore, it explores the effects of intercropping on the quality of *P. polyphylla* and elucidates the underlying mechanisms, thereby providing a theoretical foundation and technical framework for the high-efficiency cultivation of this species.

## 2 Materials and methods

### 2.1 Study location and experimental conditions

The study was conducted at the Taozhou Base, located in Guangze County, Nanping City, Fujian Province, China (27°18′N, 117°0′E, 298 meters above sea level). The climate is subtropical monsoon, with an annual accumulated temperature of 6,336 °C, total sunshine duration of 1,615 h annual precipitation of 1,864 mm, and a frost-free period of 271 days (Su et al., 2024). The experiment was carried out under the canopy of a 20 year old Chinese fir (*Cunninghamia lanceolate*) forest, providing approximately 50% shading. The soil type at the site was sandy loam.

### 2.2 Experimental design and treatments

**Figure 1:**
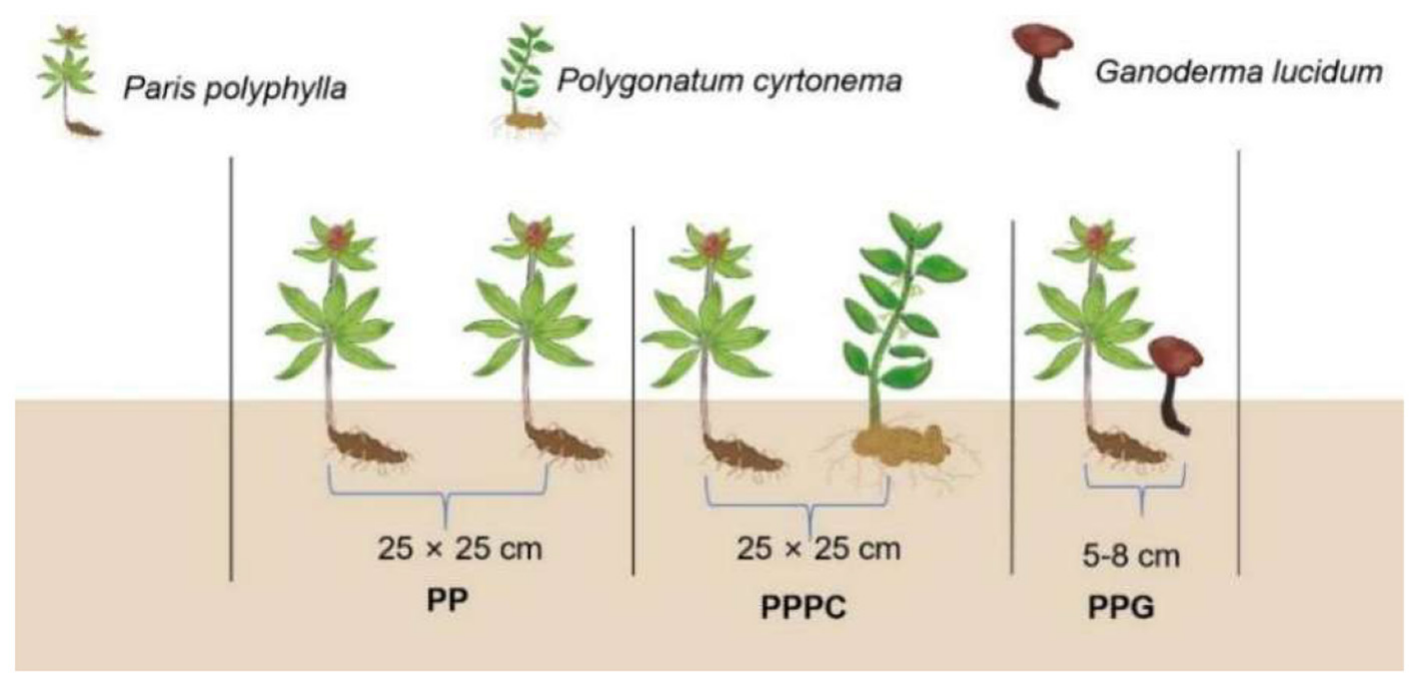
Diagram illustrating the different intercropping treatments and planting configurations used in this study. The monoculture systems of *P. polyphylla* (PP) was compared with two intercropping treatments: *P. polyphylla* with *P. cyrtonema* (PPPC) and *P. polyphylla* with *G. lucidum* (PPG). Each treatment features distinct plant spacing and root configurations, with the spacing between plants shown as 25 × 25 cm for PP and PPPC systems, while 5-8 cm distances for PPG systems.

The experiment was carried out from March 2022 to October 2024. The experimental design comprised a monoculture system of *P. polyphylla* (PP) and two intercropping treatments: *P. polyphylla* intercropped with *P. cyrtonema* (PPPC) and *P. polyphylla* intercropped with *G. lucidum* (PPG) (Figure 1). Each treatment included 100 plants, with three replicates per treatment. Seedlings of *P. polyphylla* and *P. cyrtonema* were 3 years old at transplantation, planted at a density of 25 cm between individual plants and 25 cm between rows. The *G. lucidum* mushroom sticks used in the intercropping treatments consisted of a composite substrate containing miscellaneous wood chips, corn cobs, and cottonseed materials. For intercropping, the outer membrane of the *G. lucidum* sticks was removed, and trenches were excavated 5 to 8 cm from the base of the *P. polyphylla* plants to embed the mushroom sticks.

Rhizosphere soil samples were collected in October 2024 employing the root-shaking method. For each treatment, ten plants exhibiting uniform size and growth were selected. Soil adhering to the roots was carefully brushed off using sterile brushes and placed into sampling bags. The collected soil samples were then divided into two parts: one part was immediately frozen in liquid nitrogen for 10 to 15 mins and subsequently stored at −80° C for microbial diversity analyses; the other portion was air-dried for subsequent physicochemical soil properties analysis.

The remaining rhizosphere soil samples were subjected to air-drying. Subsequently, the dried soil was ground and sieved following the soil treatment protocol outlined by Li F. Q. et al. (2025) to facilitate the analysis of soil physicochemical properties. The subterranean plant parts were carefully washed and oven-dried at 60° C. Following drying, these plant samples were ground and prepared for further analytical procedures.

### 2.3 Agronomic traits

The agronomic characteristics of *P. cyrtonema* were assessed following a 2 year cultivation period of *P. polyphylla*. In August 2024, measurements were taken for plant height, stem length, stem diameter, leaf length, leaf width, leaf count, and sepal count. Subsequently, in October 2024, additional parameters were assessed, including the fresh weight of the aboveground parts (comprising leaves, flowers, fruits, and stems), total root biomass, rhizome length and diameter, fresh rhizome weight, as well as the quantity and proportion of white roots relative to the entire root system.

### 2.4 Quantification of bioactive constituents in *P. polyphylla* rhizomes

The acid-insoluble ash content of the rhizomes was determined in accordance with the National Food Safety Standard for Ash Content in Foods. Furthermore, concentrations of polyphyllin I, II, and VII within the rhizomes were quantified following the protocols outlined in the 2020 edition of the Pharmacopeia of the People's Republic of China (Su et al., 2024). Specifically, 0.500 g of the test samplewas accurately weighed and placed into a stoppered conical flask, to which 25 mL of ethanolwas added. The flaskwas weighed, then subjected to reflux heating for 30 mins, cooled, and weighed again. The weight loss was compensated with ethanol, the mixture was thoroughly shaken and filtered to obtain test solution. Reference standards ofpolyphyllin I, polyphyllin II, and polyphyllin VII (4 mg each) were separately weighed, dissolved in 1 mL of methanol, and mixed thoroughly. Chromatographic analysis was conducted using a ZORBAX SB-C18 column (Agilent) was used, with acetonitrile (A) and water (B) as the mobile phase, maintained 25° C, a flow rate of 0.8 mL/min, and detection wavelength at 203 nm. A 10 μL aliquot of each reference standard and test solution was injected into the high-performance liquid chromatography (Agilent, 1,290 series, USA) system for quantification.

### 2.5 Soil analysis

Soil properties were analyzed following the methodologies described in the Soil Agrochemical Analysis by (Bao 2000). Soil pH was measured using a pH meter (Shanghai Yidian Scientific Instruments Co., Ltd. China), with a soil-to-water ratio of 1:25. The soil and water mixture was stirred for 5 mins and then allowed to stand for 1 h before measurement. Soil Organic Matter (SOM) content was assessed using the potassium dichromate-sulfuric acid oxidation method. Alkali-hydrolyzable nitrogen (AHN) was quantified using the Kjeldahl method. Available phosphorus (AP) was extracted using ammonium fluoride and hydrochloric acid and subsequently measured by molybdenum-antimony colorimetry. Available potassium (AK) was measured using flame photometry with an ammonium acetate solution. Total nitrogen (TN) was quantified using an automatic nitrogen analyzer. The contents of total phosphorus (TP) and total potassium (TK) contents were determined by digestion with nitric acid and perchloric acid, followed by hydrofluoric acid decomposition and dissolution in hydrochloric acid, with subsequent analysis via flame photometry.

### 2.6 Extraction of microbial DNA from soil, PCR amplification and sequencing

Microbial genomic DNA was extracted using the E.Z.N.A.^®^ Soil DNA Kit (Omega Bio-tek, Norcross, GA, USA) through a service provided by Shanghai Majorbio Biopharmaceutical Technology Co., Ltd. DNA quality and concentration were assessed via 1.0% agarose gel electrophoresis and quantified using a NanoDrop 2,000 spectrophotometer (Thermo Fisher Scientific, USA). DNA sequencing was performed using the Illumina PE300 platform. Raw sequencing data were submitted to the NCBI Sequence Read Archive (SRA) database (Li F. Q. et al., 2025).

### 2.7 Data analysis

Statistical analysis of agronomic traits, rhizosphere soil physicochemical properties, and bioactive components in *P. polyphylla* rhizomes under different intercropping systems was conducted using the Least Significant Difference (LSD) test implemented in the DPS (Data Processing System) software. Microbial diversity analyses were performed using the Majorbio cloud platform. Alpha diversity indices, including Shannon, Simpson, Ace, Chao, and Sobs, were calculated using Mothur (version v.1.30.2). Principal Component Analysis (PCA) was performed to analyze the variation in microbial community composition. Analysis of Similarities (ANOSIM) was used to test the significance of differences in community structure among groups. Redundancy analysis (RDA) was conducted to investigate relationships between soil physicochemical properties and soil micro bial communities. Pearson correlation coefficient was employed to examine the relationships between soil physical and chemical properties, root weight, rhizome fresh weight, effective components of *P. polyphylla*, and soil microorganisms at the phylum level. Visualization of data, including bar charts and heatmaps, was performed using the R programming language (version 3.3.1).

## 3 Results

### 3.1 Influence of intercropping systems on the agronomic characteristics of *P. polyphylla*

The impact of various intercropping systems on the agronomic characteristics of *P. polyphylla* exhibited variation (Table 1). Specifically, the fresh weight of rhizomes under the PPG intercropping system was significantly greater than that observed in both the PP and PPPC systems (*P* < 0.05), demonstrating a 51.30% increase relative to the PP monoculture, thereby indicating a pronounced enhancement in biomass accumulation. No statistically significant differences were detected among the three planting patterns concerning other agronomic parameters, including plant height, flower stem length, and stem thickness. Nonetheless, parameters such as leaf number, root weight, rhizome length, stem thickness, leaf length, number of white roots, and the ratio of white roots were slightly higher in the PPG system compared to the PP and PPPC systems. Notably, the proportion of white roots in the PPG system (0.72) increased by 46.90% relative to the PP system (0.49), suggesting that intercropping with *G. lucidum* may enhance nutrient uptake by promoting root vitality. In contrast, most agronomic traits measured in the PPPC system were slightly reduced compared to those in the PP system.

**Table 1:** Agronomic traits of *P. polyphylla* under different intercropping systems.

**Sample**	**Plant height/(cm)**	**Peduncle length/(cm)**	**Stem diameter/(mm)**	**Number of leaves**	**Number of sepals**	**Fresh weight of aerial parts/(g)**	**Root weight/(g)**	**Rhizome length/(mm)**	**Rhizome diameter/(mm)**	**Fresh weight of rhizome/(g)**	**Leaf length/(cm)**	**Leaf width/(cm)**	**Number of white roots**	**Proportion of white roots**
PP^1^	90.05 ± 17.23a^2^	14.85 ± 1.34a	9.00 ± 1.64a	6.50 ± 1.05a	4.50 ± 0.76a	34.16 ± 4.41a	3.70 ± 0.49a	146.74 ± 27.31a	51.33 ± 9.32a	60.91 ± 8.53b	18.80 ± 1.06a	6.48 ± 0.91a	19.20 ± 1.64a	0.49 ± 0.10a
PPPC	76.55 ± 13.83a	14.05 ± 1.53a	7.96 ± 0.71a	6.50 ± 0.55a	5.33 ± 0.52a	23.46 ± 1.97a	2.48 ± 0.37a	148.31 ± 9.10a	56.93 ± 9.43a	42.93 ± 8.00b	17.45 ± 1.29a	6.00 ± 1.19a	12.80 ± 1.17a	0.48 ± 0.10a
PPG	82.19 ± 17.82a	11.85 ± 1.42a	8.14 ± 1.3a	6.67 ± 0.82a	5.00 ± 0.81a	31.51 ± 3.85a	4.30 ± 0.73a	173.23 ± 12.10a	60.07 ± 11.83a	92.16 ± 10.06a	19.30 ± 1.57a	6.09 ± 1.67a	22.40 ± 2.41a	0.72 ± 0.13a

^1^PP, Monoculture of *P. polyphylla*; PPPC, Intercropping of *P. cyrtonema* and *P. polyphylla*; PPG, Intercropping of *P. polyphylla* and *G. lucidum*.

^2^Different lowercase letters within the same column indicate statistically significant differences (*P* < 0.05).

### 3.2 Variation in active ingredient content of *P. polyphylla* under different intercropping systems

Significant differences were observed in the acid-insoluble ash content and the concentrations of active compounds in *P. polyphylla* across the all three cultivation treatments (Table 2). The acid-insoluble ash content reached its maximum in the PPPC treatment, measuring 0.27 g/100 g, which was significantly greater than the levels recorded in the PPG and PP treatments (*P* < 0.05), representing a 350.00% increase relative to the PP monoculture. Additionally, the concentration of polyphyllin I was significantly higher in the PPG treatment compared to PPPC, although no significant difference was detected between PPG and PP (Table 2). The highest concentration of polyphyllin II (0.05%) was observed in the PPPC treatment, corresponding to a 20.00% increase compared to the PP monoculture (*P* < 0.05). Polyphyllin VII and total polyphyllin contents were greatest in the PPG treatment, at 1.03% and 1.09%, respectively, which were significantly higher than those in PPPC and PP (*P* < 0.05), reflecting increases of 34.16% and 30.60% relative to the PP monoculture (*P* < 0.05). Furthermore, the levels of polyphyllin VII and total polyphyllin in PPPC were significantly elevated compared to PP (*P* < 0.05), with increases of 1.6% and 1.7%, respectively. These results indicate that intercropping *P. polyphylla* with *G. lucidum* and *P. cyrtonema* notably enhances the accumulation of active constituents in *P. polyphylla*

**Table 2:** Content of active ingredients of *P. polyphylla* under different intercropping modes.

**Samples**	**Acid-insoluble ash (g·100g^−1^·DW)**	**Polyphyllin I (%·DW)**	**Polyphyllin II (%·DW)**	**Polyphyllin VII (%·DW)**	**Total polyphyllin (%·DW)**
PP^1^	0.06 ± 0.01c^2^	0.019 ± 0.001a	0.045 ± 0.001b	0.77 ± 0.01c	0.83 ± 0.00c
PPPC	0.27 ± 0.00a	0.012 ± 0.001b	0.054 ± 0.000a	0.78 ± 0.00b	0.85 ± 0.00b
PPG	0.15 ± 0.01b	0.020 ± 0.000a	0.037 ± 0.001c	1.03 ± 0.00a	1.09 ± 0.00a

^1^PP: Monoculture of *P. polyphylla*; PPPC, Intercropping of *P. cyrtonema* and *P. polyphylla*; PPG, Intercropping of *P. polyphylla* and *G. lucidum*.

^2^Different lowercase letters within the same column indicate statistically significant differences (*P* < 0.05).

### 3.3 Characteristics of rhizosphere soil of *P. polyphylla* under different intercropping systems

As shown in Table 3, compared to monoculture, the PPPC and PPG intercropping systems significantly enhanced soil nutrient parameters—including Soil Organic Matter (SOM), available phosphorus (AP), available potassium (AK), total nitrogen (TN), and total potassium (TK)—relative to monoculture cultivation. Specifically, the PPPC system increased these parameters by 16.93%, 4.90%, 20.67%, 11.64%, and 2.61%, respectively, whereas the PPG system exhibited increases of 2.82%, 26.78%, 50.28%, 0.87%, and 2.87%, respectively. Under the PPPC intercropping regime, SOM and TN concentrations were significantly higher than those observed in the PPG system; conversely, the contents of alkali-hydrolyzable nitrogen (AHN), AP, and AK were significantly lower in PPPC compared to PPG. No significant differences were detected in total potassium (TK) content between the two intercropping patterns. Additionally, the PPPC system significantly elevated AHN content by 4.63% while markedly reducing total phosphorus (TP) levels by 15.84%. In contrast, the PPG system significantly decreased AHN by 4.82%, with no significant variation in TP content relative to the PP monoculture. Across all three cultivation treatments, rhizosphere soil pH ranged narrowly from 6.49 to 6.52, indicating a slightly acidic environment. These results suggest that intercropping, particularly the PPG system, more effectively promotes the accumulation of key soil nutrients than monoculture.

**Table 3:** Physical and chemical properties of rhizosphere soil of *P. polyphylla* under different intercropping modes.

**Planting modes**	**pH**	**Organic matter(SOM) g·kg^−1^**	**Alkali-hydrolyzable nitrogen(AHN) mg·kg^−1^**	**Available phosphorus(AP) mg·kg^−1^**	**Available potassium(AK) mg·kg^−1^**	**Total nitrogen(TN) g·kg^−1^**	**Total phosphorus(TP) g·kg^−1^**	**Total potassium(TK) g·kg^−1^**
PP^1^	6.52 ± 0.02a^2^	47.19 ± 0.14c	159.67 ± 0.29b	51.20 ± 0.00c	93.87 ± 0.12c	2.32 ± 0.00c	1.01 ± 0.02a	35.98 ± 0.29b
PPPC	6.51 ± 0.01a	55.18 ± 0.80a	167.07 ± 0.06a	53.71 ± 0.01b	113.27 ± 0.23b	2.59 ± 0.00a	0.85 ± 0.00b	36.92 ± 0.17a
PPG	6.49 ± 0.01a	48.52 ± 0.03b	151.97 ± 0.03c	64.91 ± 0.01a	141.07 ± 0.06a	2.34 ± 0.00b	1.02 ± 0.04a	37.01 ± 0.73a

^1^PP, Monoculture of *P. polyphylla*; PPPC, Intercropping of *P. cyrtonema* and *P. polyphylla*; PPG, Intercropping of *P. polyphylla* and *G. lucidum*.

^2^Different lowercase letters within the same column indicate statistically significant differences (*P* < 0.05).

### 3.4 Characteristics of rhizosphere soil microbial communities of *P. polyphylla* under various intercropping systems

#### 3.4.1 Soil microbial diversity

In this investigation, diversity analyses conducted on nine soil samples yielded a total of 554,751 optimized fungal sequences and 529,512 optimized bacterial sequences. The fungal sequence libraries demonstrated coverage values ranging from 99.95% to 99.98%, while bacterial sequence libraries exhibited coverage between 99.80% and 99.90%, indicating comprehensive representation of microbial diversity within the rhizosphere soil. A total of 3,562 fungal Operational Taxonomic Units (OTUs) and 9,373 bacterial OTUs were identified.

**Figure 2:**
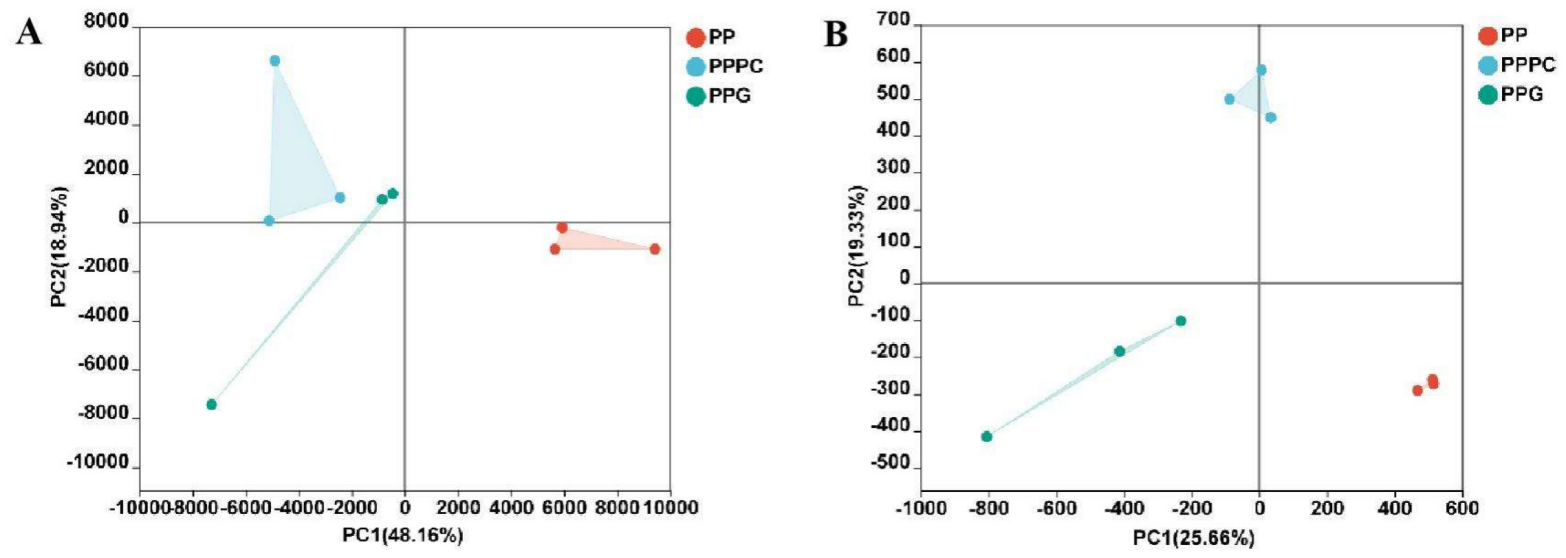
The influence of various intercropping patterns on microbial species diversity. **(A)** Fungal species diversity; **(B)** Bacterial species diversity. PP, Monoculture of *P. polyphylla*; PPPC, Intercropping of *P. cyrtonema* and *P. polyphylla*; PPG, Intercropping of *P. polyphylla* and *G. lucidum*.

Alpha diversity indices for soil fungi revealed that the Ace, Chao, and Sobs indices under the PPPC and PPG intercropping patterns were significantly higher than those observed in the PP monoculture system (*P* < 0.05) (Table 4). In contrast, the Shannon and Simpson indices did not exhibit statistically significant differences among these treatments. Although the alpha diversity indices for PPPC were marginally higher than those for PPG, this difference was not statistically significant. Principal component analysis (PCA) of fungal community composition (Figure 2A) further indicated that the fungal community structures in the rhizosphere soils of PP, PPPC, and PPG were broadly similar.

**Table 4:** Alpha diversity indices of fungal and bacterial communities in rhizosphere soil of *P. polyphylla* under different intercropping modes.

**Fungal species diversity**							**Bacterial species diversity**					
**Sample**	**Ace index**	**Chao index**	**Shannon index**	**Simpson index**	**Coverage (%)**	**Sobs index**	**Ace index**	**Chao index**	**Shannon index**	**Simpson index**	**Coverage (%)**	**Sobs index**
PP^1^	609.435 ± 47.43b^2^	608.07 ± 48.44b	4.05 ± 0.38a	0.048 ± 0.02a	99.97	606.00 ± 49.73b	1769.76 ± 79.11a	1758.84 ± 77.23a	6.858 ± 0.04a	0.00 ± 0.00a	99.83	1752.00 ± 75.03a
PPPC	758.17 ± 31.12a	756.95 ± 30.86a	4.41 ± 0.22a	0.05 ± 0.01a	99.98	755.67 ± 30.89a	1631.89 ± 74.62a	1621.72 ± 70.77a	6.79 ± 0.03a	0.00 ± 0.00a	99.87	1617.00 ± 68.02a
PPG	721.56 ± 114.04a	721.03 ± 113.72a	4.40 ± 0.60a	0.04 ± 0.04a	99.98	719.33 ± 112.08a	1643.71 ± 7.23a	1637.14 ± 5.90a	6.81 ± 0.05a	0.00 ± 0.00a	99.90	1634.00 ± 6.08a

^1^PP, Monoculture of *P. polyphylla*; PPPC, Intercropping of *P. cyrtonema* and *P. polyphylla*; PPG, Intercropping of *P. polyphylla* and *G. lucidum*.

^2^Different lowercase letters in the same column indicate statistically significant differences (*P* < 0.05).

In contrast, bacterial alpha diversity indices revealed no significant differences in the Ace, Chao, and Sobs indices among the PP, PPPC, and PPG treatments (Table 4). Notably, the Ace, Chao, Shannon, and Sobs indices were marginally higher in the PP treatment relative to PPPC and PPG. PCA revealed significant differences in bacterial community structures in the PPPC and PPG treatments differed significantly from those observed in the PP treatment (Figure 2B).

#### 3.4.2 Composition of soil microbial communities

**Figure 3:**
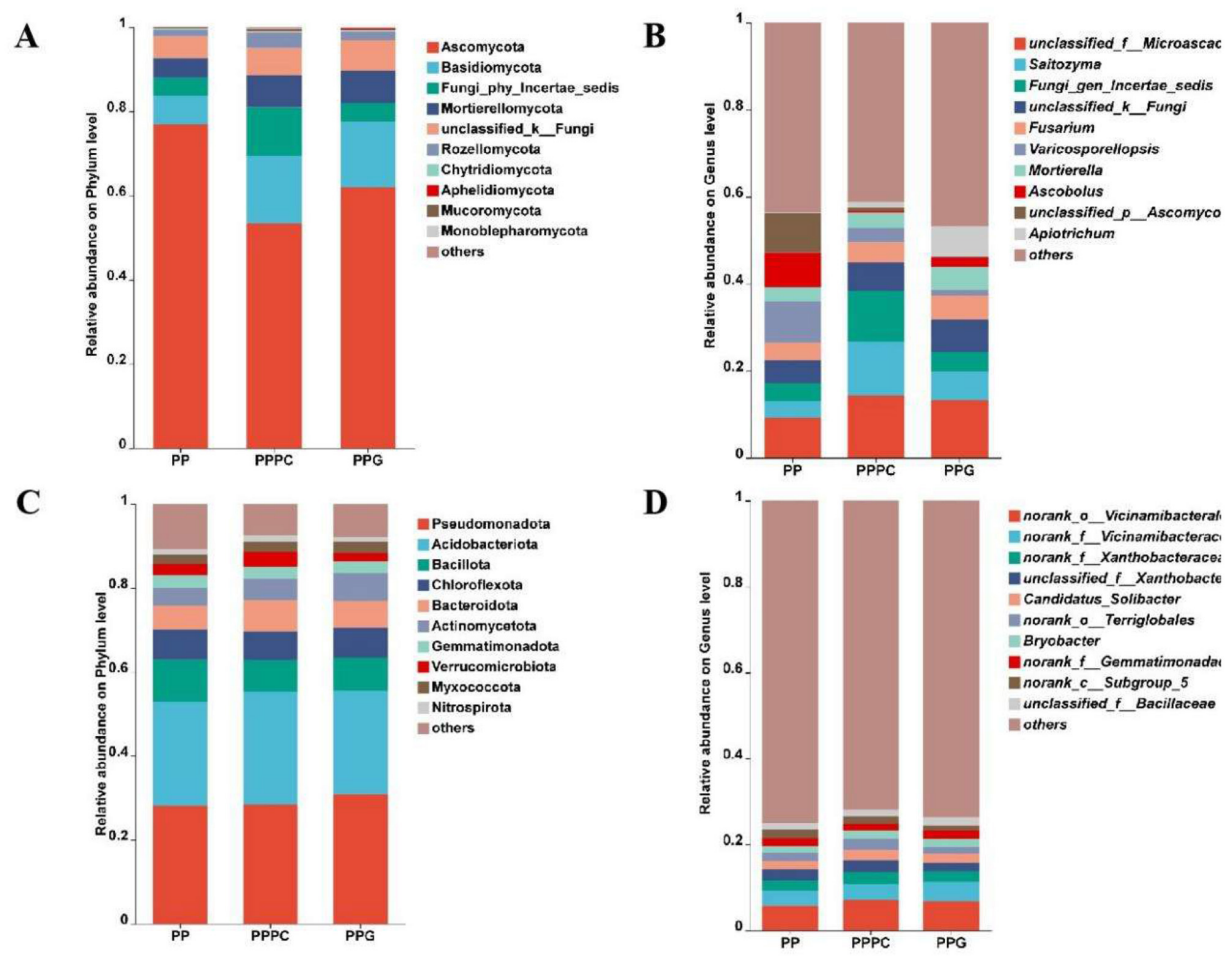
The effects of different intercropping patterns on the composition of fungal species in the rhizosphere soil of *P. polyphylla*. **(A)** Fungal phylum level; **(B)** Fungal genus level; **(C)** Bacterial phylum level; **(D)** Bacterial genus level. PP, Monoculture of *P. polyphylla*; PPPC, Intercropping of *P. cyrtonema* and *P. polyphylla*; PPG, Intercropping of *P. polyphylla* and *G. lucidum*.

Analysis identified 15 fungal phyla and 592 fungal genera across the nine soil samples. The horizontal distribution of fungal phyla within the rhizosphere soil across different intercropping systems was depicted in Figure 3A. The ten most abundant fungal phyla, ranked by relative abundance, included Ascomycota, Basidiomycota, Fungi_phy_Incertae_sedis, Mortierellomycota, unclassified_k_Fungi, Rozellomycota, Chytridiomycota, Aphelidiomycota, Mucoromycota, and Monoblepharomycota. Ascomycota and Basidiomycota predominated across all treatments, with Ascomycota accounting for 76.89%, 53.41%, and 61.97% in the PP, PPPC, and PPG models, respectively. Basidiomycota showed relative abundances of 6.85%, 15.96%, and 15.52% in the corresponding models.

At the genus level (Figure 3B), the ten most abundant fungal genera were *unclassified_f_Microascaceae* (9.25%−14.35%), *Saitozyma* (3.78%−12.45%), *Fungi_gen_Incertae_sedis* (4.22%−11.57%), *unclassified_k_Fungi* (5.28%−7.40%), *Fusarium* (3.99%−5.42%), *Varicosporellopsis* (1.41%−9.41%), *Mortierella* (3.24%−5.22%), *Ascobolus* (0.51%−8.10%), *unclassified_p_Ascomycota* (0.29%−9.00%), and *Apiotrichum* (0.15%−6.94%).

Bacterial community analysis identified 43 bacterial phyla and 907 bacterial genera within the same soil samples. At the phylum level (Figure 3C), the ten most abundant bacterial phyla were Pseudomonadota, Acidobacteriota, Bacillota, Chloroflexota, Bacteroidota, Actinomycetota, Gemmatimonadota, Verrucomicrobiota, Myxococcota, and Nitrospiraota. Pseudomonadota and Acidobacteriota dominated across all treatments, with relative abundances of 28.05%, 28.50%, and 30.84% for Pseudomonadota and 24.74%, 26.87%, and 24.69% for Acidobacteriota in PP, PPPC, and PPG, respectively.

At the genus level (Figure 3D), the ten most abundant bacterial genera included *norank_o_Vicinamibacterales* (5.76%−7.12%), *norank_f_Vicinamibacteraceae* (3.54%−4.46%), *norank_f_Xanthobacteraceae* (2.31%−2.76%), *unclassified_f_Xanthobacteraceae* (1.93%−2.70%), *Candidatus_Solibacter* (2.04%−2.39%), *norank_o_Terriglobales* (1.51%−2.52%), *Bryobacter* (1.55%−1.96%), *norank_f_Gemmatimonadaceae* (1.69%−1.84%), *norank_c_Subgroup_5* (1.31%−2.04%), and classified_f_Bacillaceae (1.51%−1.90%).

#### 3.4.3 Variations in soil microbial community composition

**Figure 4:**
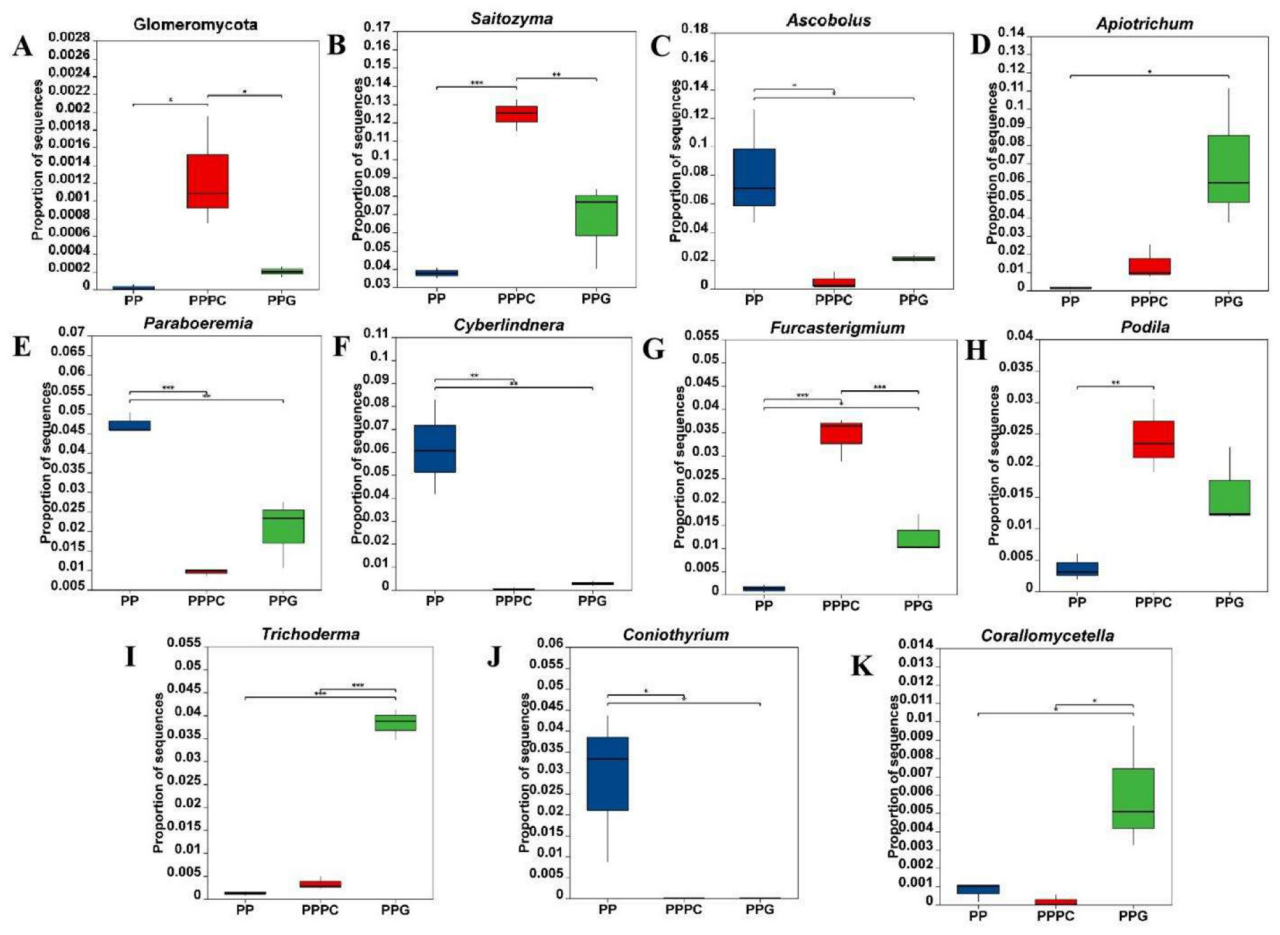
Relative abundance changes of differential fungi in the rhizosphere soil of *P. polyphylla* under different intercropping patterns. **(A)** Phylum level; **(B-K)** Genus level. PP, Monoculture of *P. polyphylla*; PPPC, Intercropping of *P. cyrtonema* and *P. polyphylla*; PPG, Intercropping of *P. polyphylla* and *G. lucidum*. * indicates a significant difference between the two groups (*P < 0.05; **P < 0.01; ***P < 0.001).

At the fungal phylum level (Figure 4A), the relative abundance of Glomeromycota in the rhizosphere soil of *P. polyphylla* under the PPPC intercropping model was markedly greater than that observed in the PP and PPG models, with an increase of 6,298% relative to the PP model (*P* < 0.05). At the fungal genus level, 61 genera demonstrated statistically significant differences in relative abundance (*P* < 0.05) across the three cultivation patterns. The ten most differentially abundant fungal genera were selected for detailed analysis (Figures 4B–K). Compared to the PP model, both the PPPC and PPG models significantly decreased the relative abundance of the genera *Ascobolus, Paraboremia, Cyberlindnera*, and *Coniothyrium*. Within the PPPC model, the relative abundances of *Saitozyma, Furcasterigium*, and *Podila* in the rhizosphere soil were significantly elevated compared to PP, with increases of 229%, 2,612%, and 565%, respectively. Additionally, the abundances of *Saitozyma* and *Furcasterigium* were significantly higher in PPPC than in PPG. Conversely, in the PPG model, the genera *Apiotrichum, Trichoderma*, and *Corallomycetella* exhibited significantly greater relative abundances than in PP, increasing by 4,529%, 3,022%, and 697%, respectively; moreover, the abundances of *Trichoderma* and *Corallomycetella* were significantly higher than those observed in PPPC. These results indicate that distinctly influence the composition and abundance of specific fungal taxa in the rhizosphere.

**Figure 5:**
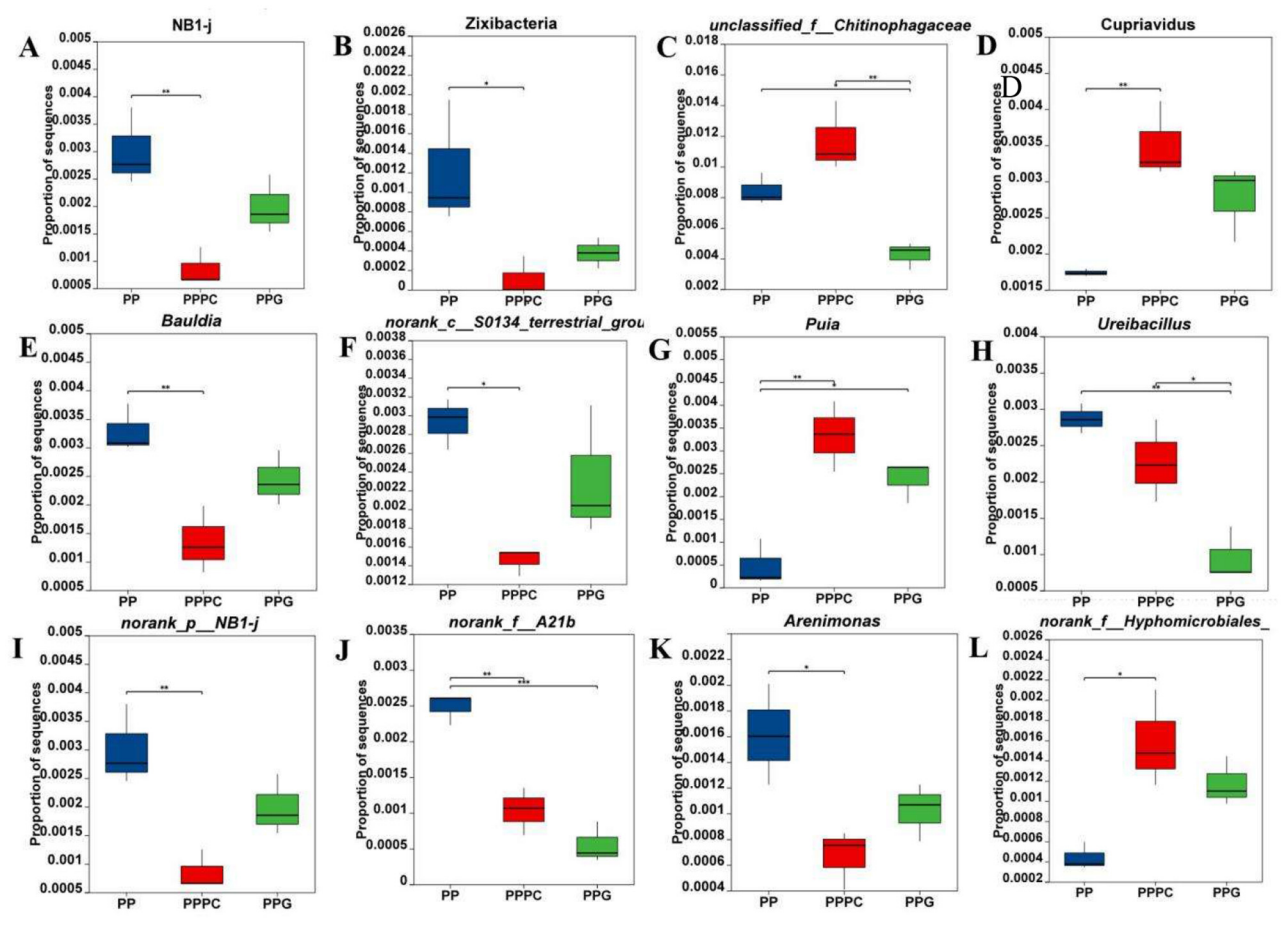
Changes in relative abundance of differential bacteria in rhizosphere soil of *P. polyphylla* under different intercropping modes **(A, B)** Phylum level; **(C-L)** Genus level. PP, Monoculture of *P. polyphylla*; PPPC, Intercropping of *P. cyrtonema* and *P. polyphylla*; PPG, Intercropping of *P. polyphylla* and *G. lucidum*. * indicates a significant difference between the two groups (*P < 0.05; **P < 0.01; ***P < 0.001).

At the bacterial phylum level (Figures 5A, B), the relative abundances of NB1-j and Zixibacteria in the rhizosphere soil of *P. polyphylla* were significantly lower in the PPPC intercropping system compared to PP. At the genus level, 58 bacterial genera exhibited significant differences in relative abundance (*P* < 0.05) across the three planting regimes in the rhizosphere soil of *P. polyphylla*. The top ten differentially abundant genera were selected for further analysis (Figures 5C–L). Compared to PP, the PPPC intercropping mode significantly increased the relative abundances of *Cupriavidus* and *norank_f__Hypomicrobiales* by 101% and 260%, respectively. Concurrently, the relative abundances of *Bauldia, norank_c__S0134_terrestrial-group, norank_p__NB1-j*, and *Arenimonas* were significantly decreased. Both PPPC and PPG intercropping modes markedly enhanced the relative abundance of *Puia* by 393% and 591%, respectively, while reducing *norank_f__A21b*. Additionally, under the PPG mode, the relative abundances of *unclassified_f__Chitinophagaceae* and *Ureibacillus* were significantly lower than those observed in PP and PPPC. These findings demonstrate that distinct intercropping patterns can modulate the composition and abundance of specific bacterial taxa within the rhizosphere soil of *P. polyphylla*.

#### 3.4.4 Analysis of the relationship between soil microbial diversity and soil physicochemical properties

**Figure 6:**
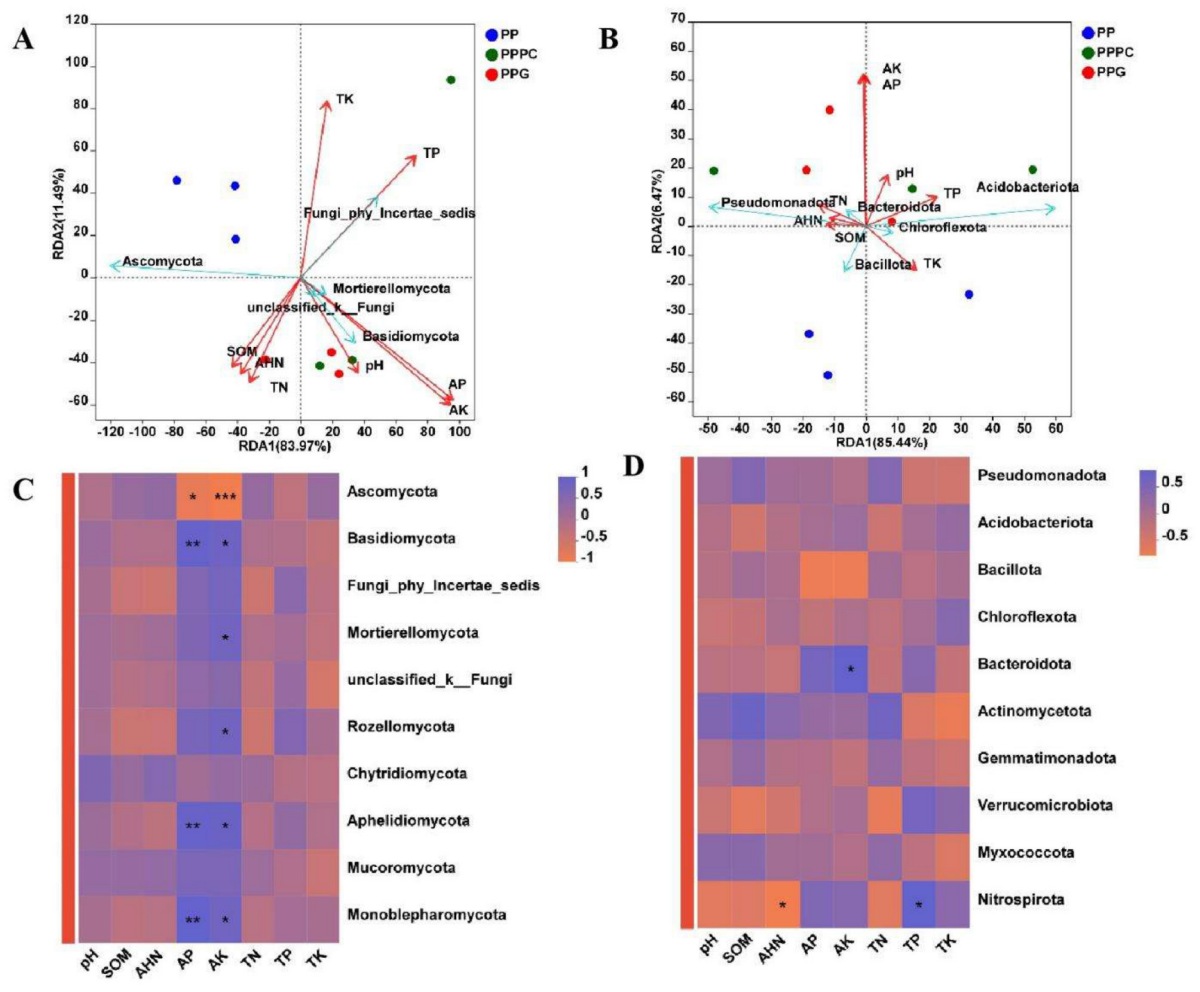
Analysis of the relationship between soil microbial community composition and soil physical and chemical characteristics. **(A, C)**: Fungal phylum level; **(B, D)**: Bacterial phylum level. PP, Monoculture of *P. polyphylla*; PPPC, Intercropping of *P. cyrtonema* and *P. polyphylla*; PPG, Intercropping of *P. polyphylla* and *G. lucidum*; SOM, Organic matter; AHN: Alkali-hydrolyzable nitrogen; AP, Available phosphorus; AK, Available potassium; TN, Total nitrogen; TP, Total phosphorus; TK, Total potassium. * indicates a significant difference between the two groups (*P < 0.05; **P < 0.01; ***P < 0.001).

Redundancy analysis (RDA) of the top ten microorganisms exhibiting the highest relative abundance in rhizosphere soil demonstrated that the combined variance explained by the first two RDA axes accounted for 95.46% at the fungal community level (Figure 6A). Soil physicochemical parameters significantly influenced the relative abundances of fungal taxa such as Ascomycota, Basidiomycota, and Fungi_phy_Incertae_sedis (Figure 6C). Similarly, for bacterial communities, the first two RDA axes explained 91.91% of the total variance, and soil physicochemical factors exerted a notable influence on the relative abundance of bacterial phyla such as Pseudomonadota, Acidobacteriota, and Bacillota (Figure 6B). Both RDA and Pearson correlation analyses indicated that the relative abundances of dominant fungal and bacterial phyla were predominantly affected by soil environmental variables, with available potassium (AK) and available phosphorus (AP) contents identified as particularly influential (Figures 6B, D).

### 3.5 Analysis of the relationship between soil microbial diversity and the quality of *P. polyphylla* across various intercropping systems

**Figure 7:**
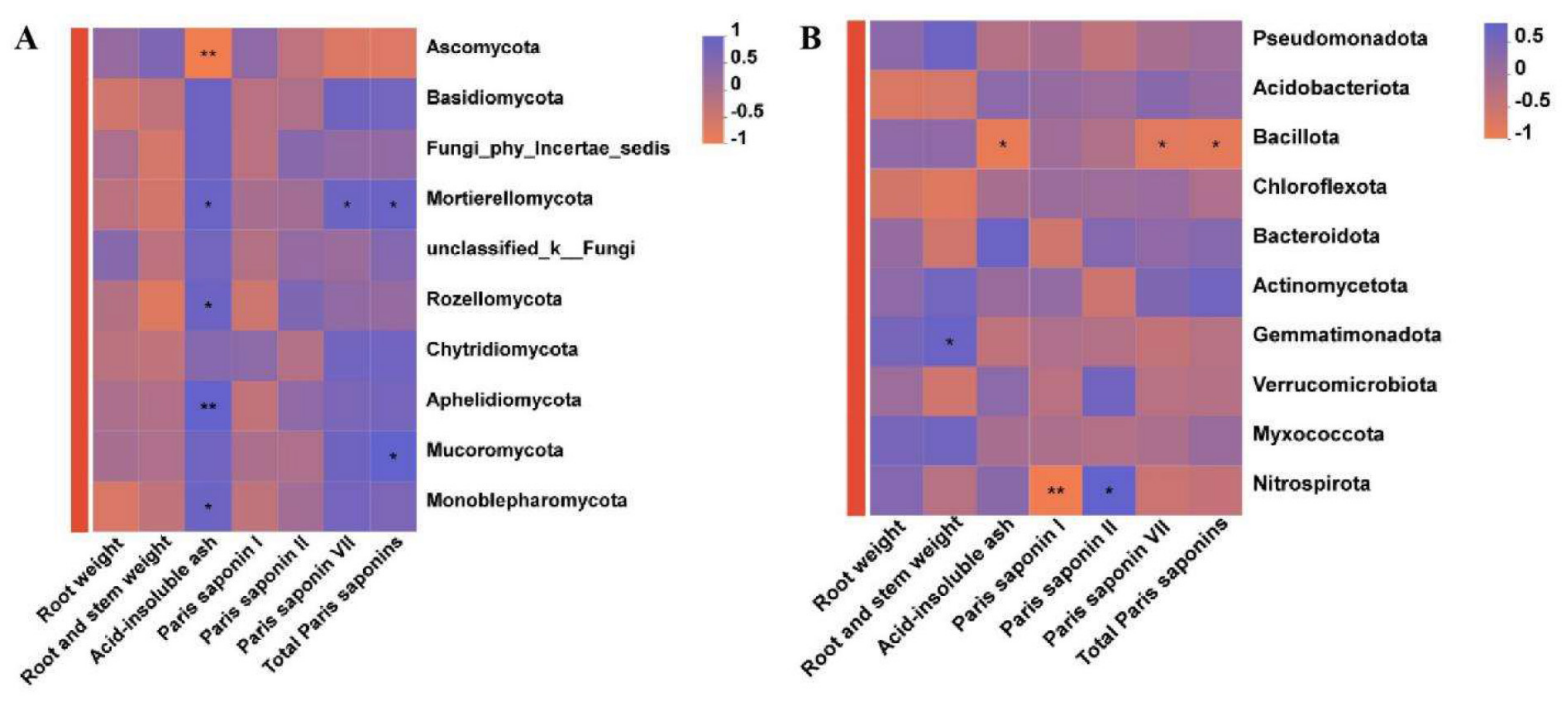
Correlation between the active components of *P. polyphylla* and the dominant soil microorganism phyla. **(A)** Components of *P. polyphylla* and dominant fungal phyla; **(B)** Components of *P. polyphylla* and dominant bacterial phyla. * indicates a significant difference between the two groups (*P < 0.05; **P < 0.01.

A correlation analysis was performed to examine the associations between the yield and active ingredient content of *P. polyphylla* under different intercropping patterns and the relative abundances of the top ten dominant fungal and bacterial phyla present in the soil. The acid-insoluble ash content of *P. polyphylla* demonstrated a highly significant negative correlation with the abundance of Ascomycota (*R* = −0.8815, *P* < 0.01), while showing significant positive correlations with Mortierellomycota (*R* = 0.7120), Rozellomycota (*R* = 0.6781), Aphelidiomycota (*R* = 0.8001), and Monoblepharomycota (*R* = 0.6809). Furthermore, polyphyllin VII (*R* = 0.6833) and total polyphyllin content (*R* = 0.7120) exhibited notable positive correlations with the Mortierellomycota phylum (Figure 7A). A significant positive correlation was also identified between the total polyphyllin content in *P. polyphylla* and the Nitrospiraota phylum (*R* = 0.7967). Regarding biomass, the fresh weight of the *P. polyphylla* rhizome was significantly positively correlated with Gemmatimonadota (*R* = 0.6667, *P* < 0.05). Conversely, acid-insoluble ash content (*R* = −0.7628), polyphyllin VII (*R* = −0.6667), and total polyphyllin content (*R* = −0.7120) were significantly negatively correlated with Bacillota. Additionally, polyphyllin I was strongly negatively correlated with Nitrospiraota (*R* = −0.8530), whereas polyphyllin II showed a strong positive correlation with Nitrospiraota (*R* = 0.7500) (Figure 7B).

## 4 Discussion

### 4.1 Impact of various intercropping patterns on the quality of *P. polyphylla*

Different intercropping configurations influence the growth and quality of medicinal plants by modulating interspecific resource allocation and microenvironmental conditions (Machiani et al., 2018; Fallah et al., 2018; Zhang et al., 2024; Ma et al., 2017). In the present study, both the *P. polyphylla*–*P. cyrtonema* (PPPC) and *P. polyphylla*–*G. lucidum* (PPG) intercropping systems enhanced the quality of *P. polyphylla*, a phenomenon closely associated with interspecific niche complementarity inherent in these patterns (Wang Q. C. et al., 2024). Both *P. polyphylla* and *P. cyrtonema* exhibit shade tolerance. However, *P. polyphylla* demonstrates a higher potassium requirement, whereas *P. cyrtonema* preferentially assimilates nitrogen (Su et al., 2024). This differentiation in nutrient assimilation reduces interspecific competition, leading to a 4.6% increase in soil alkali-hydrolyzable nitrogen (AHN) under the PPPC system. This nutrient enrichment underpins the synthesis of acid-insoluble ash (linked to mineral accumulation) and polyphyllin II, whose contents increased by 350.00% and 20.00%, respectively. In the PPG system, *G. lucidum*, functioning as a beneficial fungus, secretes cellulase and lignin peroxidase (Zhou et al., 2018), facilitating the decomposition of insoluble organic matter into small-molecule carbon sources, which was evidenced by a 2.8% increase in soil organic matter (SOM). This process supplies energy for polyphyllin biosynthesis in *P. polyphylla*. Concurrently, the available phosphorus (AP) content, elevated by 26.78% relative to monoculture (PP), promotes the accumulation of polyphyllin VII and total polyphyllin by enhancing the activity of key enzymes in the mevalonate pathway, such as 3-hydroxy-3-methylglutaryl-CoA reductase (Cho et al., 2022). Consequently, polyphyllin VII and total polyphyllin contents increased by 34.16% and 30.60%, respectively, compared to PP. These species-specific regulatory mechanisms suggest that intercropping patterns can be strategically selected based on desired active compounds: PPPC is preferable for augmenting acid-insoluble ash, whereas PPG is advantageous for enhancing total polyphyllin content.

### 4.2 Influence of intercropping patterns on soil physicochemical properties associated with *P. polyphylla*

Alterations in soil nutrient content constitute a critical determinant of plant growth, and appropriate intercropping practices can modify soil physicochemical characteristics (Lan et al., 2025). This investigation suggested that soil nutrient levels under both intercropping patterns (PPPC and PPG) were significantly increased compared to monoculture (PP). Specifically, PPPC markedly improved SOM and AK, whereas PPG was most effective in enhancing AP and AK. These findings align previous report that intercropping medicinal plants beneath forest canopies improves soil physicochemical properties and fertility by establishing a synergistic “plant-microbe” nutrient transformation network (Zhang et al., 2016). The 2.82% increase in SOM observed in PPG is primarily attributable to the biological characteristics of *G. lucidum*, including the composition and decomposition of its fungal bag and the release of mycelial metabolites into the soil (Cen et al., 2024). As a wood-decaying fungus with potent lignocellulose-degrading capabilities, *G. lucidum* mycelia extend into the fungal bag and adjacent soil, secreting extracellular enzymes such as cellulase, ligninase, and hemicellulase (Ematou et al., 2025; Zhou et al., 2013). Moreover, *G. lucidum* promotes mineralization of native soil organic matter via the “priming effect” (Zhang et al., 2017), which indirectly enhances AP by 26.78% and AK by 50.28%. In addition, the PPPC cultivation pattern demonstrated the highest AHN content, potentially attributable to flavonoids secreted by *P. cyrtonema* roots that promote the proliferation of nitrogen-fixing bacteria, such as Hyphomicrobiales (Wu et al., 2025). This process increases soil nitrogen availability, thereby providing an additional nitrogen source for *P. polyphylla*. The enrichment of specific microbial taxa, including *Saitozyma*, within the rhizosphere of *P. polyphylla* contributes to the improvement of soil structure and the enhancement of nutrient cycling (Bastida et al., 2016), thereby fostering a more conducive growth environment for *P. cyrtonema*. This microbially mediated resource complementarity mechanism mitigates interspecific resource competition and facilitates mutualistic interactions between the two species (Han et al., 2025). Further investigation of this “plant–nitrogen-fixing bacteria” interaction through metagenomic sequencing is warranted to substantiate these findings.

### 4.3 Effects of intercropping patterns on rhizosphere soil microbial communities of *P. polyphylla*

Soil microbial communities are integral to nutrient cycling and plant–microbe interactions, with community structure predominantly influenced by environmental factors such as intercropping regimes and soil physicochemical properties (Li et al., 2024; Wang R. Y. et al., 2024; Liu Y. et al., 2025). This study demonstrated that intercropping systems induced more pronounced alterations in rhizosphere microbial diversity and composition than monoculture, with fungal communities exhibiting a stronger response than bacterial communities. Arbuscular mycorrhizal fungi within the Glomeromycota phylum, known for forming symbiotic associations that enhance nutrient uptake and stress tolerance (Abarca et al., 2024), showed significantly increased relative abundance under the PPPC system. Additionally, genera such as *Saitozyma* (Basidiomycota), *Furcasterigium* (Ascomycota), and *Podila* (Mortierellomycota), which contribute to soil organic matter decomposition and pathogen suppression (Bastida et al., 2016; Liu H. et al., 2025; Zhao et al., 2020), were enriched under PPPC. These taxa contribute to the accumulation of soil nitrogen, phosphorus, and potassium, thereby promoting healthy plant development. Within the PCG intercropping system, *Trichoderma* demonstrates capabilities for solubilizing phosphorus and potassium, in addition to exhibiting a synergistic interaction with nitrogen-fixing bacteria (Qin et al., 2024). Consequently, the PPPC and PCG intercropping models enhance soil nutrient availability by optimizing the functional groups of fungi.

At the bacterial genus level, PPPC intercropping significantly increased the relative abundance of *Cupriavidus, norank_f_Hyphomicrobiales*, and *Puia*. *Cupriavidus*, through its metabolic activities, may enhance soil phosphorus availability, indirectly facilitating plant phosphorus uptake (Wang, 2023). *Hyphomicrobiales* encompasses various rhizobia, including *Rhizobiaceae* strains capable of symbiotic nitrogen fixation with legumes (Volpiano et al., 2021), indicating that PPPC also augments soil nitrogen fixation capacity. *Puia*, a Gram-negative, aerobic, rod-shaped, non-motile bacterium (Lv et al., 2017), exhibited increased abundance in both PPPC and PPG systems. Furthermore, research has demonstrated that crops can modulate the rhizosphere microbial communities of adjacent plants through interspecific interactions. For instance, root exudates from potato onion (*Allium cepa* var. *agrogatum* Don.), particularly flavonoids such as taxifolin, have been shown to modify the chemical profile of tomato (*Solanum lycopersicum*) root exudates, thereby facilitating the recruitment of beneficial bacteria, including those belonging to the genus *Bacillus* (Zhou et al., 2023; Zhu et al., 2025). Similarly, root exudates from ginger, which contain compounds such as sinapyl alcohol and 6-gingerol, can induce chrysanthemum roots to secrete specific metabolites that enhance the proliferation and colonization of *Burkholderia* species within the chrysanthemum rhizosphere (Zhu et al., 2025). Therefore, variations in microbial community composition among intercropping patterns may be attributable to differences in root exudate profiles of *P. cyrtonema, P. polyphylla*, and *G. lucidum*, a hypothesis that could be further examined through exudate compositional analyses.

### 4.4 Influence of rhizosphere microbial community structure on the quality of *P. polyphylla*

The modulation of medicinal herb quality via intercropping fundamentally represents a cascade process involving soil nutrients, microorganisms, and secondary metabolites (Gong et al., 2021; Duan et al., 2024). Correlation analyses in this study confirmed that specific microbial taxa are closely associated with the accumulation of active compounds, underscoring the pivotal role of functional microorganisms as mediators. Central to this cascade is the capacity of microorganisms to link soil nutrient dynamics with plant secondary metabolism through their metabolic activities (Bettina et al., 2025; Bikash et al., 2018). For instance, the fungal phylum Mortierellomycota exhibited positive correlations with polyphyllin VII and total polyphyllin content in *P. polyphylla*, suggesting potential secretion of enzymes that facilitate polyphyllin biosynthesis. Bacterial taxa also contributed to quality regulation. The abundance of Gemmatimonadota was positively correlated with rhizome fresh weight, while the abundance of Bacillota was negatively correlated with acid-insoluble ash, polyphyllin VII, and total polyphyllin. These findings imply that intercropping not only enriches beneficial microbial populations but may also suppress potentially deleterious groups, thereby maintaining a balanced quality formation process. This research extends current understanding of microbial regulation of medicinal plant quality (Wang R. Y. et al., 2024) and provides a foundation for optimizing intercropping strategies through targeted microbial management. Various intercropping configurations have been shown to significantly elevate the abundance of *Trichoderma* populations. The proliferation of this microbial group has been demonstrated to reduce the incidence of crop disease (Xu et al., 2022) while concurrently enhancing crop yield substantially (Szczech et al., 2024). Moreover, during crop cultivation, the deliberate inoculation of *Trichoderma* not only achieves these benefits but also markedly increases the accumulation of flavonoids and terpenoids within the crops (Kulbat-Warycha et al., 2024). These findings further corroborate the role of *Trichoderma* in modulating plant secondary metabolic pathways and promoting the biosynthesis of secondary metabolites (Jurić et al., 2020). In the present investigation, the PPG intercropping pattern was found to enrich *Trichoderma* populations and augment the accumulation of polyphyllin I and VII in *P. polyphylla*, suggesting that *Trichoderma* may facilitate polyphyllin biosynthesis in this species; however, additional experimental validation is warranted in future studies.

## 5 Conclusions

The results indicate that the PPPC intercropping pattern (*P. polyphylla—P. cyrtonema*) enriches the phylum Glomeromycota and the genera *Saitozyma* and *Cupriavidus*, thereby enhancing soil nitrogen and phosphorus cycling and promoting the accumulation of acid-insoluble ash and polyphyllin II. This pattern is particularly suitable for cultivation scenarios aiming to balance mineral content with specific saponin profiles. Conversely, the PPG intercropping pattern (*P. polyphylla*—*G. lucidum*) leverages the mycelial network of *G. lucidum* to increase available potassium, activate fungal taxa such as Trichoderma, and significantly elevate levels of polyphyllin VII and total saponins through synergistic interactions involving the Chaetomiaceae family and associated metabolic pathways. This configuration is optimal for cultivation objectives prioritizing total saponin content. Collectively, this study elucidates the directional relationships between rhizosphere microbial communities and target phytochemical components under diverse intercropping regimes of medicinal plants, thereby providing a theoretical framework to address continuous cropping challenges in the cultivation of rare medicinal species. Practically, intercropping patterns can be selected based on desired phytochemical targets, or their efficacy can be enhanced through the artificial inoculation of functional microorganisms.

## Data Availability

The original contributions presented in the study are publicly available. This data can be found here: https://www.ncbi.nlm.nih.gov/, accession number PRJNA1347261.

## References

[B1] Abarca C. Bidondo L. F. Bompadre J. Velázquez M. S. (2024). Arbuscularmycorrhizal fungi in tomato tolerance to pathogens and nematodes: a comprehensive review. Sci. Hortic. 329:112969. doi: 10.1016/j.scienta.2024.112969

[B2] Bao S. D. (2000). Soil and Agricultural Chemistry Analysis, 3rd Edn. Beijing:China Agriculture Press.

[B3] Bastida F. Torres I. F. Moreno J. L. Baldrian P. Ondoño S. Ruiz-Navarro A. . (2016). The active microbial diversity drives ecosystem multifunctionality and is physiologically related to carbon availability in Mediterranean semi-arid soils. Mol. Ecol. 25, 4660–4467. doi: 10.1111/mec.13783 27481114

[B4] Bettina B.e, M. Marco A. N. Mariangela H. (2025). Microbial secondary metabolites and their use in achieving sustainable agriculture: present achievements and future challenges. Agronomy. 15, 1350–1350. doi: 10.3390/agronomy15061350

[B5] Bikash B. Amir A. Mikko M. K. (2018). Activation of microbial secondary metabolic pathways: avenues and challenges. Synth. Syst. Biotechnol. 3, 163–178. doi: 10.1016/j.synbio.2018.09.001 30345402 PMC6190515

[B6] Cen Q. Fan J. Zhang R. Chen H. Y. Hui F. Y. Li J. M. . (2024). Impact of *Ganoderma lucidum* fermentation on the nutritional composition, structural characterization, metabolites, and antioxidant activity of Soybean, sweet potato and Zanthoxylum pericarpium residues. Food Chem. X. 21:101078. doi: 10.1016/j.fochx.2023.101078 38205161 PMC10776642

[B7] Chen Y. L. Guo Y. Q. Han S. J. Zou C. J. Zhou Y. M. Cheng G. L. (2002). Effect of root derived organic acids on the activation of nutrients in the rhizosphere soil. J. For. Res.13, 115–118. doi: 10.1007/BF02857233

[B8] Cho S. H. Tóth K. Kim D. Vo P. H. Lin C. H. Handakumbura P. P. . (2022). Activation of the plant mevalonate pathway by extracellular ATP. Nat. Commun. 13, 450–450. doi: 10.1038/s41467-022-28150-w 35064110 PMC8783019

[B9] Duan W. Y. Chen X. L. Ding Y. Mao X. Y. Song Z. j. . (2024). Intricate microbe-plant-metabolic remodeling mediated by intercropping enhances the quality of Panax quinquefolius L. Physiol. Plant. 176:e14499. doi: 10.1111/ppl.14499 39221485

[B10] Ematou N. L. N. Njouonkou A. L. Moundipa F. P. (2025). Assessment of extracellular enzymes in mycelial culture of some fungi from the Western highlands of Cameroon. J. Mater. Environ. Sci. 16, 328–340. doi: 10.1016/j.funbio.2025.08.012

[B11] Fallah S. Rostaei M. Lorigooini Z. Surki A. A. (2018). Chemical compositions of essential oil and antioxidant activity of dragonhead (Dracocephalum moldavica) in sole crop and dragonhead-soybean (Glycine max) intercropping system under organic manure and chemical fertilizers. Ind. Crops Prod. 115, 158–165. doi: 10.1016/j.indcrop.2018.02.003

[B12] Fang L. X. Li J. C. Chen X. X. Xu X. Q. (2024). How lignocellulose degradation can promote the quality and function of dietary fiber from bamboo shoot residue by Inonotus obliquus fermentation. Food Chem. 451:139479. doi: 10.1016/j.foodchem.2024.139479 38696939

[B13] Feng S. F. Wu L. J. Guan Y. M. Wang Z. Q. Wang Y. P. (2008). Inhibition effects of volatile oil of asarum on 4 kinds of pathogens of ginseng using in vitro tests. Special Wild Econ. Anim. Plant Res. 37–39. doi: 10.16720/j.cnki.tcyj.2008.03.003

[B14] Gong X. Y. Shi J. B. Zhou X. G. Yuan T. Gao D. M. Wu F. Z. (2021). Crop rotation with cress increases cucumber yields by regulating the composition of the rhizosphere soil microbial community. Front. Microbiol. 12:631882. doi: 10.3389/fmicb.2021.631882 33776961 PMC7994511

[B15] Han M. Zhang Z. Yang H. Du J. Wu X. Fu Y. (2025). The effect of intercropping with eucommia ulmoides on the growth and quality of abelmoschus manihot and its rhizosphere microbial community. Agronomy 15:863. doi: 10.3390/agronomy15040863

[B16] Jurić S. Sopko S. K. Król-Kilińska Ż. Žutić I. Uher S. F. Ðermić E. . (2020). The enhancement of plant secondary metabolites content in Lactuca sativa L. by encapsulated bioactive agents. Sci. Rep. 10:3737. doi: 10.1038/s41598-020-60690-3 32111947 PMC7048752

[B17] Kulbat-Warycha K. Nawrocka J. Kozłowska L. Żyżelewicz D. (2024). Effect of light conditions, trichoderma fungi and food polymers on growth and profile of biologically active compounds in *Thymus vulgaris* and *Thymus serpyllum*. Int. J. Mol. Sci.. 25:4846. doi: 10.3390/ijms25094846 38732065 PMC11084565

[B18] Lan X. Liang Z. H. Yang H. X. Li H. R. Ruan L. X. Wei W. L. . (2025). Effects of sugarcane and *Platostoma palustre* intercropping on soil physicochemical properties and crop yield. *Crops*. 1–9. [Epub ahead of print].

[B19] Li B. Li Y. Y. Wu H. M. Zhang F. F. Li C. J. Li X. X. . (2016). Root exudates drive interspecific facilitation by enhancing nodulation and N2 fixation. Proc. Natl. Acad. Sci. USA. 113, 6496–6501. doi: 10.1073/pnas.1523580113 27217575 PMC4988560

[B20] Li F. Q. Liu S. Z. Luo G. S. Zou Y. L. Huang W. Zeng M. S. (2025). Analysis of bacterial community structure and diversity in rhizosphere soil of *Monochasma savatieri* in different habitats. Sci. Silvae Sin. 61, 47–56. doi: 10.11707/j.1001-7488.LYKX20240002

[B21] Li M. L. Chen X. B. Cui Y. S. Yue X. Qi L. F. Huang Y. L. . (2025). Mechanism of soil microbial community degradation under long-term tomato monoculture in greenhouse. Front. Microbiol. 16:1587397. doi: 10.3389/fmicb.2025.1587397 40800111 PMC12339526

[B22] Li Y. Ding Z. Xu T. Wang Y. L. Wu Q. L. Song T. J. . (2024). Synthetic consortia of four strains promote Schisandra chinensis growth by regulating soil microbial community and improving soil fertility. Planta. 259, 135–135. doi: 10.1007/s00425-024-04410-5 38678496

[B23] Liu H. Ma Z. H. Liu W. F. Wan M. H. Ma F. L. Wu N. . (2025). Effects of different tillage practices with organic fertilizers on rhizosphere soil microbial communities of maize in saline - alkali soils. Chin. J. Eco-Agric. 33, 25–39. doi: 10.12357/cjsa.20240308

[B24] Liu L. L. Ding M. Y. Xie Q. Luo W. B. Chen Q. X. Su H. L. (2025). Genetic diversity analysis of *Paris polyphylla* Sm. and construction of primary core germplasm. Fujian J. Agric. Sci. 40, 234–244. doi: 10.19303/j.issn.1008-0384.2025.03.003

[B25] Liu Y. Fan Y. Kuzyakov Y. Dai J. Zhang C. Li C. (2025). Effects of maize/soybean intercropping on nitrogen mineralization and fungal communities in soil. Plant Soil 1-15. doi: 10.1007/s11104-025-07824-6

[B26] Lu Y. P. Gao Z. Zhu Y. L. Mao J. P. Yao D. L. Wang X. L. (2025). Construction and evaluation of *Polygonatum cyrtonema* Hua intercropping basedon the growth and physiological adaptability. Plant Sci. J. 43, 253–264. doi: 10.11913/PSJ.2095-0837.24100

[B27] Lv Y. Y. Gao Z. H. Xia F. Chen M. H. Qiu L. H. (2017). Puia dinghuensis gen. *nov., sp. nov.*, isolated from monsoon evergreen broad-leaved forest soil. Int. J. Syst. Evol. Microbiol. 67:46. doi: 10.1099/ijsem.0.002346 28984557

[B28] Ma Y. H. Fu S. L. Zhang X. P. Zhao K. Chen H. Y. H. (2017). Intercropping improves soil nutrient availability, soil enzyme activity and tea quantity and quality. Agric. Ecosyst. Environ. Appl. Soil Ecol. 119, 171–178. doi: 10.1016/j.apsoil.2017.06.028

[B29] Machiani M. A. Javanmard A. Morshedloo M. R. Filippo M. (2018). Evaluation of yield, essential oil content and compositions of peppermint (Mentha piperita L.) intercropped with faba bean (Vicia faba L.). J. Clean. Prod.. 171, 529–537. doi: 10.1016/j.jclepro.2017.10.062

[B30] Qin L. Gao Y. Wang L. Ran J. Ou X. Wang Y. . (2024). Trichoderma consortium compost corncob promotes the growth of Pseudostellaria heterophylla and alleviates its diseases incidence via reshaping the soil microbiome and improving the soil nutrients under continuous monocropping conditions. Ind. Crops Prod. 216:118800. doi: 10.1016/j.indcrop.2024.118800

[B31] Rao Z. W. Sun Y. Y. Guo J. J. Jin H. Yang Z. P. Zhang Q. (2025). A review on the continuous cropping obstacles of rhizomatous medicinal plants. Soil Fertil. Sci. China. 4, 258–272. doi: 10.11838/sfsc.1673-6257.24469

[B32] Su H. L. Zheng M. X. Chen H. Zhu Y. M. Zhu Y. J. (2024). Optimum nitrogen, phosphorus and potassium combination increasesthe rhizome yield and saponin content of *Paris polyphylla*. J. Plant Nutr. Fertil. 30, 128–136. doi: 10.11674/zwyf.2023279

[B33] Szczech M. Kowalska B. Wurm F. R. Ptaszek M. Boncela A. J. Trzciński P. . (2024). The effects of tomato intercropping with medicinal aromatic plants combined with trichoderma applications in organic cultivation. Agronomy 14, 2572–2572. doi: 10.3390/agronomy14112572

[B34] Volpiano C. G Sant'A F H. Ambrosini A. De S. J. J. F. B. Beneduzi A. Whitman W. B. . (2021). Genomic metrics applied to rhizobiales (Hyphomicrobiales): species reclassification, identification of unauthentic genomes and false type strains. Front. Microbiol. 12:614957. doi: 10.3389/fmicb.2021.614957 33841347 PMC8026895

[B35] Wang H. X. (2023). Effect of organic fertilizer on soil, leaf nutrition, fruit quality and microbial community in citrus orchards. (MS Thesis of Southwest University, Chongqing). 1–82. doi: 10.27684/d.cnki.gxndx.2023.003493

[B36] Wang Q. C. Peng X. J. Yuan Y. X. Zhou X. D. Huang J. Q. Wang H. N. (2024). The effect of Torreya grandis inter-cropping with *Polygonatum sibiricum* on soil microbial community. Front. Microbiol. 15:1487619. doi: 10.3389/fmicb.2024.1487619 39697655 PMC11652488

[B37] Wang R. Y. Liu C. Y. Bie X. S. Dai Y. Feng X. Wang R. . (2024). Pecan-medicinal crops intercropping improved soil fertility and promoted interactions between soil microorganisms and metabolites. Chem. Biol. Technol. Agric. 11,162–162. doi: 10.1186/s40538-024-00693-8

[B38] Wei F. H. Zhang J. X. Zhou D. P. Xu Y. H. Gong J. Z. Zhao L. (2024). Effect of ginseng-asarum strip cropping on the soil microhabitat of ginseng rhizosphere. SSRN. 35:ssrn4789910. doi: 10.2139/ssrn.4789910

[B39] Wu J. Liu S. Zhang H. Chen S. Si J. Liu L. . (2025). Flavones enrich rhizosphere Pseudomonas to enhance nitrogen utilization and secondary root growth in Populus. Nat. Commun. 16:1461. doi: 10.1038/s41467-025-56226-w 39920117 PMC11805958

[B40] Wu Z. Zhang J. Xu F. R. Wang Y. Z. Zhang J. Y. (2017). Rapid and simple determination of polyphyllin I, II, VI, and VII in different harvest times of cultivated *Paris polyphylla* Smith var. yunnanensis (Franch.) Hand.-Mazz by UPLC-MS/MS and FT-IR. J. Nat. Med. 71, 139–147. doi: 10.1007/s11418-016-1043-8 27665608

[B41] Xian K. Su J. Fu C. He W. Liu B. Xie D. . (2022). Microbial diversity in rhizosphere soil of *Paris polyphylla* var. chinensis in different growth years. in Chinese. Guihaia 42, 2087–2098. *guihaia.gxzw*202103004

[B42] Xu H. Yan L. Zhang M. Chang X. Zhu D. Wei D. . (2022). Changes in the density and composition of rhizosphere pathogenic fusarium and beneficial trichoderma contributing to reduced root rot of intercropped soybean. Pathogens 11, 478–478. doi: 10.3390/pathogens11040478 35456153 PMC9031213

[B43] Yang J. M. Wu Q. H. Wang Y. T. Chen X. Y. Gao W. Zhao Y. . (2023). Suppression of banana fusarium wilt disease with soil microbial mechanisms via pineapple rotation and residue amendment. Agronomy 13, 377–377. doi: 10.3390/agronomy13020377

[B44] Zhang C. Liu G..B. Xue S. Wang G. L. (2016). Soil bacterial community dynamics reflect changes in plant community and soil properties during the secondary succession of abandoned farmland in the Loess Plateau. Soil Biol. Biochem. 97, 40–49. doi: 10.1016/j.soilbio.2016.02.013

[B45] Zhang R. Li X. C. Qu J. X. Zhang D. D. Cao L. X. Qin X. M. . (2024). Intercropping with maize and sorghum-induced saikosaponin accumulation in Bupleurum chinense DC. by liquid chromatography-mass spectrometry-based metabolomics. J. Mass Spectrom. 59, e5035–e5035. doi: 10.1002/jms.5035 38726730

[B46] Zhang X. W. Han X. Z. Yu W. T. Wang P. Cheng W. X. (2017). Priming effects on labile and stable soil organic carbon decomposition: pulse dynamics over two years. PLoS ONE 12:e0184978. doi: 10.1371/journal.pone.0184978 28934287 PMC5608328

[B47] Zhao X. H. Chai S. S. Zhang M. M. Fan Y. C. Mao Y. F. Mao Z. Q. . (2020). Effects of shell powder on microbial diversity in acidified soil and growth of malus hupehensis var. mengshanensis seedlings. Sci. Silvae Sin. 56, 153–163. doi: 10.11707/j.1001-7488.20200917

[B48] Zhou S. Zhang J. S. Ma F. Y. Tang C. H. Tang Q. J. Zhang X. Y. (2018). Investigation of lignocellulolytic enzymes during different growth phases of *Ganoderma lucidum* strain G0119 using genomic, transcriptomic and secretomic analyses. PLoS ONE 13:e0198404. doi: 10.1371/journal.pone.0198404 29852018 PMC5979026

[B49] Zhou X. Zhang J. Khashi U. R. M. Gao D. Wei Z. Wu F. . (2023). Interspecific plant interaction via root exudates structures the disease suppressiveness of rhizosphere microbiomes. Mol. Plant. 16, 849–864. doi: 10.1016/j.molp.2023.03.009 36935607

[B50] Zhou X. W. Cong W. R. Su K. Q. Zhang Y. M. (2013). Ligninolytic enzymes from Ganoderma spp: current status and potential applications. Crit. Rev. Microbiol. 39, 416–426. doi: 10.3109/1040841X.2012.722606 22992227

[B51] Zhu L. Zhou W. Wang J. Guo J. Zhou C. (2025). Root exudate-mediated assemblage of rhizo-microbiome enhances Fusarium wilt suppression in chrysanthemum. Microbiol. Res. 292:128031. doi: 10.1016/j.micres.2024.128031 39705829

